# The Role of Augmented Reality Neuronavigation in Transsphenoidal Surgery: A Systematic Review

**DOI:** 10.3390/brainsci13121695

**Published:** 2023-12-08

**Authors:** Benedetta Maria Campisi, Roberta Costanzo, Vincenzo Gulino, Chiara Avallone, Manfredi Noto, Lapo Bonosi, Lara Brunasso, Gianluca Scalia, Domenico Gerardo Iacopino, Rosario Maugeri

**Affiliations:** 1 Neurosurgical Clinic, AOUP “Paolo Giaccone”, Post Graduate Residency Program in Neurologic Surgery, Department of Biomedicine Neurosciences and Advanced Diagnostics, School of Medicine, University of Palermo, 90127 Palermo, Italy; benedettamaria.campisi@community.unipa.it (B.M.C.); vincenzo.gulino@community.unipa.it (V.G.); chiara.avallone@community.unipa.it (C.A.); manfredi.noto@community.unipa.it (M.N.); lapo.bonosi@community.unipa.it (L.B.); lara.brunasso@community.unipa.it (L.B.); gerardo.iacopino@gmail.com (D.G.I.); rosario.maugeri1977@gmail.com (R.M.); 2 Neurosurgery Unit, Department of Head and Neck Surgery, Garibaldi Hospital, 95122 Catania, Italy; gianluca.scalia@outlook.it

**Keywords:** augmented reality, transsphenoidal surgery, endoscopy, microscopic surgery

## Abstract

In the field of minimally invasive neurosurgery, microscopic transsphenoidal surgery (MTS) and endoscopic transsphenoidal surgery (ETS) have been widely accepted as a safe approach for pituitary lesions and, more recently, their indications have been extended to lesions at various skull base regions. It is mandatory during transsphenoidal surgery (TS) to identify key anatomical landmarks in the sphenoid sinus and distinguish them from the lesion. Over the years, many intraoperative tools have been introduced to improve the neuronavigation systems aiming to achieve safer and more accurate neurosurgical interventions. However, traditional neuronavigation systems may lose the accuracy of real-time location due to the discrepancy between the actual surgical field and the preoperative 2D images. To deal with this, augmented reality (AR)—a new sophisticated 3D technology that superimposes computer-generated virtual objects onto the user’s view of the real world—has been considered a promising tool. Particularly, in the field of TS, AR can minimize the anatomic challenges of traditional endoscopic or microscopic surgery, aiding in surgical training, preoperative planning and intra-operative orientation. The aim of this systematic review is to analyze the potential future role of augmented reality, both in endoscopic and microscopic transsphenoidal surgeries.

## 1. Introduction

Augmented reality (AR), also referred to as ‘Enhanced Reality’ or simply ‘Image Enhancement’ (IE), is advanced technology that superimposes computer-generated virtual objects onto the user’s view of the real world. Unlike virtual reality (VR), which immerses the user in an entirely computer-generated environment, AR aims to enhance a real-world image with virtual objects or subjects [1,2,3].

**Figure 1:**
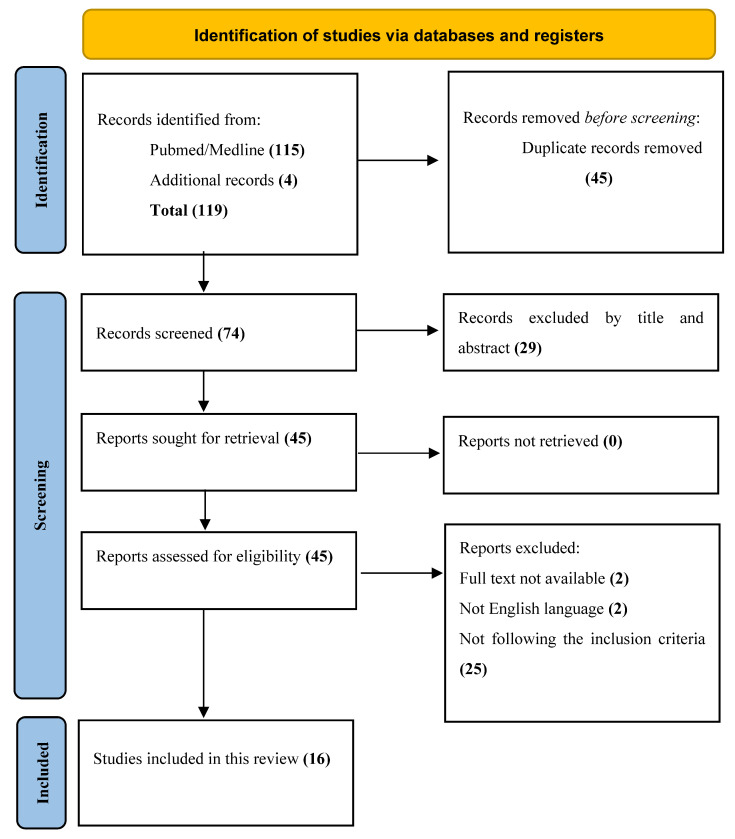
PRISMA flow diagram.

In the age of minimally invasive surgery (MIS), where multiple critical structures are involved in a remarkably compact anatomical region and landmarks identification is critical, the use of such advanced technology has been considered a promising tool to improve visualization and surgical guidance since it was first described in the clinical setting by Kelly et al. [4] and Roberts et al. [5] in the 1980s. In the field of MIS, microscopic transsphenoidal surgery (MTS) and, more recently and more widely used, transsphenoidal endoscopic endonasal surgery (ETS) [5], are among the most commonly performed minimally invasive neurosurgical procedures for the resection of pituitary lesions (Figure 1) [6,7,8,9]. MTS and especially ETS have proven to be safe and effective, guarantying a good post-operative outcome. Despite the obvious post-operative advantages of minimally invasive surgery, especially when compared to open transcranial surgical approaches (OTCS), their limited visualization remains a critical hurdle that has not yet been adequately overcome by standard surgical neuronavigation guidance systems. 

To deal with this hurdle, an AR guidance system may have several roles and applications in TS surgery. AR provides real-time information overlaid on the surgeon’s view, which can include 3D reconstructions of the surgical field, anatomical landmarks and tumor boundaries. This enhanced visualization may improve the surgeon’s spatial orientation and accuracy during the neurosurgical procedure. In addition, AR can be used as a navigation guidance system that overlays preoperative imaging data directly onto the patient’s anatomy, allowing the surgeon to follow a planned path for tumor resection even in cases where landmarks are obscured or absent [9]. Moreover, for patients with significant anatomical variants, especially those with conditions like acromegaly [8,10,11,12,13], AR can adapt to these variations by constantly updating the surgical plan based on real-time patient-specific data, ensuring precision and safety.

The aim of this systematic review is to analyze the potential future role of AR in both endoscopic and microscopic TS: could AR navigation be the next promising tool in the evolution of minimally invasive transsphenoidal surgery?

## 2. Materials and Methods

We conducted a systematic review, according to the Preferred Reporting Items for Systematic Reviews and Meta-Analyses (PRISMA) [14] (Figure 1) guidelines, of the literature using PubMed/Medline, and additional articles were identified through manual searches on PubMed, Google Scholar and Scopus. Ethical approval and patient consent were not required for this study.

**Figure 2:**
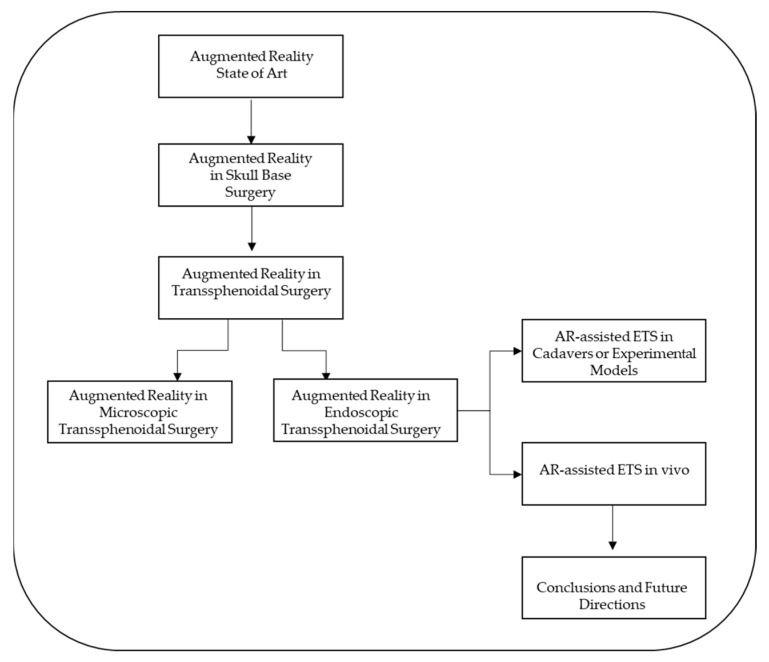
A flow chart of the systematic review’s key stages.

Herein, a flow chart of the systematic review’s key stages is reported (Figure 2).

The following Mesh Terms were used:

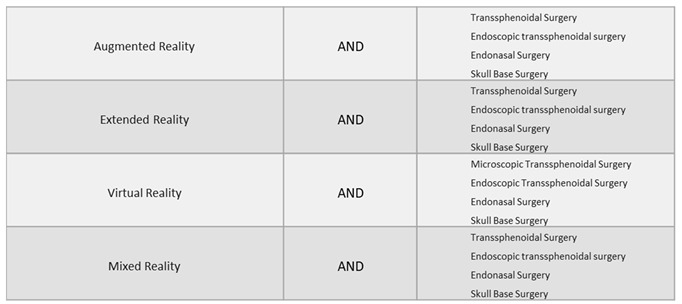



Two independent reviewers performed the study selection to include all relevant papers from the literature. Collected data were evaluated by 2 authors (V.G. and C.A). Disagreement was solved via discussion and consensus, with a third author mediating (B.M.C.) whenever necessary. Duplicated papers were removed using Microsoft Excel 16.37 Software (Redmond, WA, USA). Then, the titles and abstracts of the search results were screened, and non-related articles were excluded. Full-text articles were assessed for eligibility. 

### 2.1. Eligibility Criteria

Articles were considered eligible according to the following criteria:

- Full article in English;
- Original peer-reviewed articles reporting the use of AR-assisted transsphenoidal surgery;
- Studies including patients (in vivo), cadaver specimens or experimental models (i.e., phantoms);
- Clinical articles: case reports, case series, prospective and retrospective cohort studies, original articles and technical notes.

### 2.2. Exclusion Criteria

- Articles not in English;
- Articles reporting surgical simulation just in virtual reality (VR).

The article’s full text was retrieved for further investigations when the title and abstract met the inclusion criteria, while irrelevant papers were excluded. Papers were selected with no time limit, including studies from 2002 to 2023.

### 2.3. Data Extraction

After selecting the relevant studies, according to the inclusion/exclusion criteria above, the extracted data from each paper were: the first author, publication year, study design, patients’ characteristics, test subjects (vivo, experimental model, cadaver specimens), pathology, AR Display Devices, AR technique, landmark identification, accuracy results, surgical time of AR-assisted surgery, reoperation or anatomical variants (ex. acromegaly), outcomes, surgeon’s perspective on using AR-assisted surgery (Table 1).

## 3. Results

A total of 119 published studies were identified using PubMed and additional reference list searches. After removing duplicates in Excel, *n* = 74 articles were screened. Based on the titles and abstracts, we then excluded 29 articles. Two articles were not retrieved because the full text was not available, two articles were excluded because they were not in the English language. Another 25 articles were excluded because they did not meet our eligibility criteria. Finally, 16 papers were included in this systematic review. 

### 3.1. Patients’ Characteristics and Demographics

**Figure 3:**
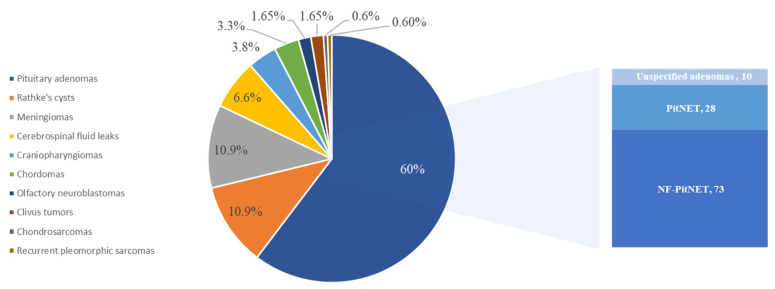
Report of the most reported pathologies.

Patients’ characteristics, demographics and treatment information were not consistently or uniformly reported across the included studies, due to the presence of cadaver dissection studies and experimental models (i.e., phantoms). Indeed, nine studies were carried out in vivo, three were cadaver dissections, three studies were experimental models, which usually used a skull phantom, and the last study used both cadaver dissection and a skull phantom. Of a total of 635 test subjects, 618 patients were evaluated (17 cadavers dissections). Of the patients, in 325 cases, gender was not specified (52.6%), 154 patients were males (24.9%) and 139 were females (22.5%) with a male/female ratio of 1.107; the mean age was 54.42 (from a range of 10–85 years old). There were 184 main neurosurgical pathologies treated, of which 111 were pituitary adenomas (60%)—73 NF-PitNET, 28 PitNET and 10 were unspecified adenomas—20 were Rathke’s cysts (10.9%), 20 meningiomas (10.9%), 12 cerebrospinal fluid leaks (6.6%), 7 craniopharyngiomas (3.8%), 6 chordomas (3.3%), 3 olfactory neuroblastomas (1.65%), 3 clivus tumors (1.65%), 1 chondrosarcoma (0.6%) and 1 was a recurrent pleomorphic sarcoma (0.6%) (Figure 3).

### 3.2. Augmented Reality Techniques

During the procedures analyzed, 15 studies used AR-assisted navigation, of which 11 studies used AR-assisted endoscopy with AR superimposed onto the endoscopic display (68.75%), 3 studies used AR-assisted microscopy (18.75%)—of which two used a heads-up microscopic display and one used AR superimposed onto the microscope eyepiece—while in one study (6.25%) AR assisted both endoscopy and microscopy. The last study (6.25%) used AR for preoperative simulation, which was then compared to intraoperative live endoscopic images.

### 3.3. Landmarks Identification

**Figure 4:**
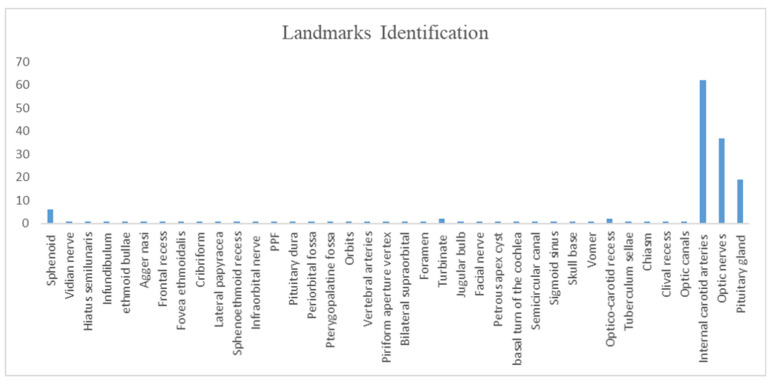
Report of landmarks identification.

Regarding the number and type of structures identified, AR-assisted neuronavigation allowed for the identification of the main anatomical landmarks in most studies, such as the internal carotid arteries (62%), the optic nerves (37%) and the pituitary gland (19%) (Figure 4).

### 3.4. Accuracy of the Results

**Figure 5:**
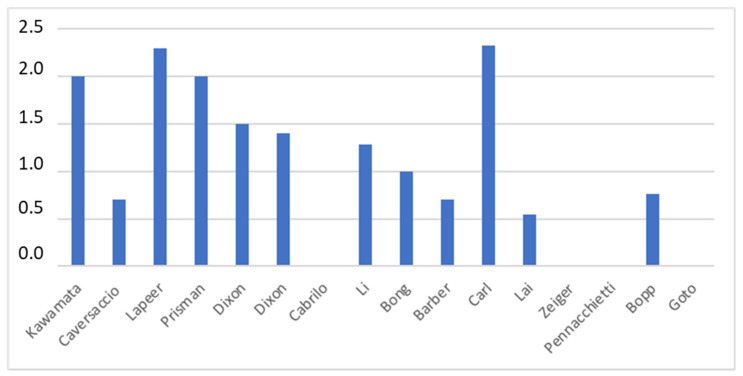
Histogram showing the target registration error (TRE) in millimeters in the reported studies.

A variety of target registration error (TRE) parameters were reported in the included studies. The average precision of the overlay was 1.37 mm with a standard deviation of 0.65 mm. The TRE was less than 2 mm in the majority of studies [15,16,17,18,19,21,22,23,24,25]. Two studies did not capture quantitatively the accuracy of the system, but surgeons have found AR-assisted navigation to be very helpful in effectively targeting lesions. [26,27] Lai et al. and Caversaccio et al. [16,25] have reported the best accuracy of the target (0.55 mm ± 0.24 DS, 0.7 mm, respectively). Goto et al. [29] showed that AR-assisted navigation was more useful than traditional navigation systems, reporting an average score overall of 4.7 (IC 95%) (Figure 5).

### 3.5. AR Surgeons’ Perspectives

Thirteen studies have shown the significant benefits and positive experiences of using AR-assisted navigation in transsphenoidal surgery. Indeed, Kawamata et al. [15] and Barber et al. [23] highlighted the benefits of knowing the exact surgical orientations, which meant surgeons were able to (i) adequately identify the details of the endoscopic surgery and (ii) to avoid delicate structures. Five studies [17,24,27,28,29] have found AR-assisted navigation to be significantly useful for all surgical tasks. In addition, four studies [19,21,22,24] have unanimously reported a significant reduction in mental effort and frustration compared to conventional neuronavigation systems. Moreover, Cabrilo et al. [20] have pointed out that AR-assisted navigation demanded less mental effort to merge the whole-time neuronavigational data with the surgical field. 

## 4. Discussion

### 4.1. Augmented Reality in Neurosurgery 

Augmented reality (AR), also referred to as ‘Enhanced Reality’ or simply ‘Image Enhancement’ (IE), is advanced technology that superimposes computer-generated virtual objects on the user’s view of the real world [1]. Unlike virtual reality (VR), which immerses the user in an entirely computer-generated environment, AR aims to enhance a real-world image with virtual objects or subjects. [1,2,3] Furthermore, mixed reality (MR) combines VR and AR by projecting virtual and responsive objects into the real world. Although the term ‘Virtual Reality’ is always distinguished from ‘Augmented Reality’, the terms ‘Mixed Reality’ and ‘Augmented Reality’ are sometimes used interchangeably [30].

In the modern scientific age, the application of these latest advanced technologies, which allow the augmentation or virtual representation of real images by creating an immersive virtual world defined as the “metaverse”, is becoming even more common in the medical field, including in neurosurgery [31]. Indeed, the term “neuroverse” is currently commonly used to describe several roles of such advanced technology in the neurosurgical field, including surgical training, preoperative planning, and intra-operative guidance [3].

Particularly, the intraoperative use of AR as an adjunct to conventional neuronavigation systems is increasingly being explored. Although the use of conventional neuronavigation systems represents a widespread and indispensable tool to modern neurosurgical practice, these systems still have several limitations. First, they display 3D patient imaging data on a 2D display, requiring the surgeon to mentally merge the data to the patient’s anatomy [30]. Secondly, these systems repeatedly divert the surgeon’s attention from the surgical field, contributing to increased fatigue and operative times, while introducing potential errors related to task-switching [30]. An AR-assisted neuronavigation system, overlaying preoperative and intraoperative patient images directly onto the patient’s anatomy, has the potential to overcome these limitations, significantly decreasing the need to shift the focus away from the surgical procedure [5].

#### 4.1.1. Can Augmented Reality Improve the Accuracy of Image-Guided Surgery?

**Figure 6:**
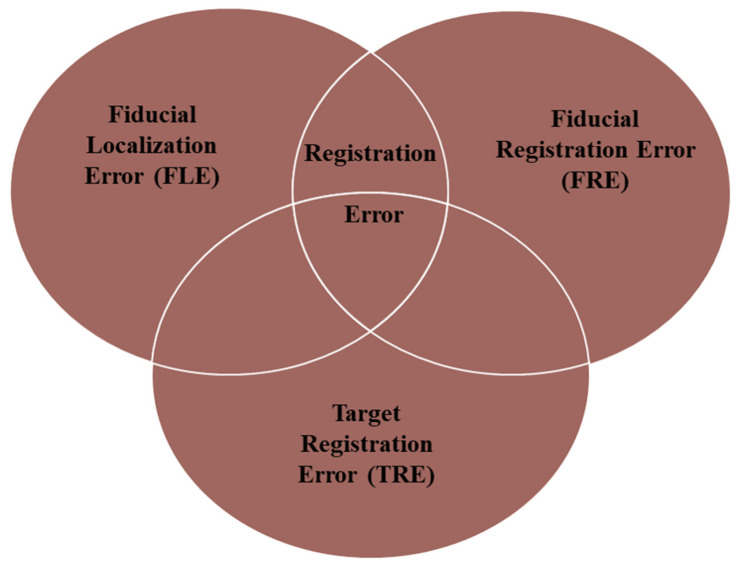
Types of Registration Errors. (1) The FLE is a random, unknown error that occurs both in the location of fiducials on a CT scan and in the location of fiducials on the patient in the operating room. (2) The FRE is the distance between the measured position of the fiducial in one space and its counterpart in another space after registration. (3) The TRE is the distance between corresponding points other than the fiducial points after registration [33,34].

Image-guided surgery (IGS) systems are currently used in neurosurgical practice, allowing a real-time, intraoperative tracking of the current position of the lesion and/or anatomical features based on preoperative images. In order to use these systems safely during surgery, it is essential to understand the registration error associated with each system to determine the level of confidence that can be placed in it. In this field, Maurer et al. [32] proposed three useful error measurements for analyzing the accuracy of point-based registration methods (Figure 6).

The TRE is the best estimate of navigation accuracy, which defines the relationship between the instrument tip and its measured position. Predictably, the lower the TRE is, the higher the accuracy is. In this field, the general consensus is that the TRE for surgical navigation should be 2 mm or less; moreover, the TRE goal stated by Citardi et al. [35,36] for a next-generation surgical navigation platform would be 1.0–1.5 mm, ideally 0.6–1.0 mm, especially in ETS, where several critical structures are involved in a remarkably compact anatomical region, and landmark identification is critical. Labadie et al. [33], in a comprehensive analysis of the conventional IGS systems commonly used in ETS, found that conventional neuronavigation systems did not consistently achieve a clinically acceptable TRE. Furthermore, even when a TRE of 2 mm is achieved, these systems may be considered as a complement to but not replacement of the knowledge of surgical anatomy. Indeed, Synderman et al. [37] prospectively recorded the TRE measured using the Stryker Navigation System (Kalamazoo, MI, USA) during 50 endoscopic anterior skull base procedures in an academic university setting. The mean error of initial registration was 2.8 mm (range: 1.4 to 7.1 mm). Thus, they attempted to achieve greater accuracy by excluding fiducials with large errors and, after excluding two fiducials, their mean final registration error decreased significantly to 1.6 mm, even though large variability remained (range: 0.6 to 3.7 mm). Registration is the process by which the IGS computer matches the preoperative images to the intraoperative surgical volume; in this context, AR navigation systems overlaying 3D-generated images directly onto the endoscopic view have been shown to (i) enable navigation while visualizing sub-surface anatomy and (ii) in some cases significantly reduce TRE, thereby improving the neuronavigation workflow. Li et al. [21] and Bong et al. [22] provided clear examples of this. They have both reported a registration accuracy with a mean TRE of less than 1.30 mm. Even more impressive were the results obtained by Lai et al. 25], who achieved a TRE of 0.55 ± 0.24 mm in CBCT image projection, with a median of 0.51 mm and an interquartile range of 0.39–0.68 mm. Nevertheless, it is essential to point out that all the mean TREs reported in the articles included in this systematic review are related to cadavers or experimental models. Thus, the accuracy of the AR navigation systems in clinical practice may be slightly different, requiring further, deeper studies. 

#### 4.1.2. Augmented Reality-Assisted Neuronavigation: Workflow

**Figure 7:**
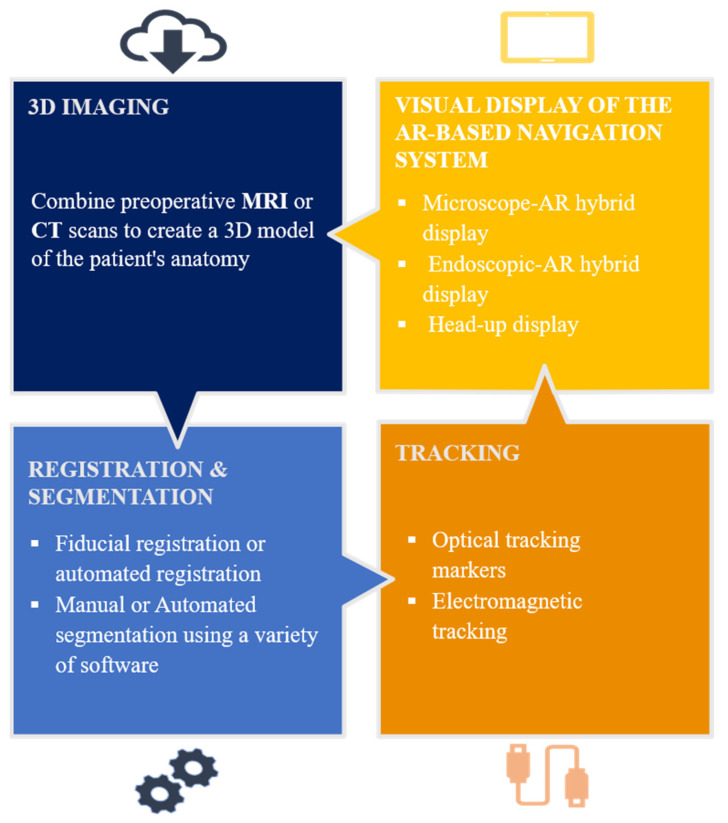
Augmented Reality Workflow. (1) 3D Imaging: combine preoperative MRI, CT or Angiography scans to create a 3D model of the patient’s anatomy. This model serves as the foundation for AR visualization. (2) Registration & Segmentation: generally fiducial or automated registration and segmentation. (3) Tracking: utilize a tracking system to continuously monitor the position and orientation in real time. This can be achieved using optical tracking markers or electromagnetic tracking. (4)Visual Display of the AR-based Navigation System: heads-up display (HUD) to the microscope eyepiece or a separate screen; 3D virtual images are superimposed onto endoscopic or microscopic live images.

In order to display AR images intra-operatively, most systems required patients’ preoperative assessment images (CT, MRI or angiographic scans), which typically involved the automatic or manual segmentation of anatomical structures from the patient’s imaging data and the use of 3D modeling software to format the images for the AR system [30]. The 3D image data is then superimposed on the real patient’s anatomical structures, and it can be viewed directly through the eyepiece of the microscope, a separate endoscopic or microscopic screen, headset displays, HoloLens smart glasses, a tablet or smartphones. A tracking system, either electromagnetic or optical, is usually used to continuously monitor the position and orientation in real time (Figure 7).

### 4.2. Augmented Reality-Assisted Neuronavigation in Transsphenoidal Surgery (TS)

TS is actually considered superior to conventional OTCS approaches due to more favorable post-operative outcomes and less collateral tissue damage due to the shorter operative route and smaller surgical site [11,30,38]. Despite the increasingly precise aim of less-invasive surgery, the technical issues related to intraoperative anatomical assessments and potential complications have largely lasted out. In this field, the use of AR-assisted neuronavigation-based TS might overcome these limitations, improving anatomy identification and intraoperative guidance.

#### 4.2.1. Augmented Reality’s Application in Microscopic Transsphenoidal Surgery (MTS)

Microscope-based AR was introduced to the neurosurgical community through surgical microscopes such as the robotic multiple-coordinate manipulator microscope (Zeiss, Oberkochen, Germany) with an integrated heads-up display (HUD), and became commercially available in the mid-1990s [24,39,40,41]. Currently, modern surgical microscopes combined with AR-assisted navigation systems are expanding their application in different cranial neurosurgical approaches [24]. As soon as these innovative surgical microscopes became clinically available, they were applied to skull base surgery, particularly transsphenoidal ones [30], as experienced by Carl et al. [24]. 

TS using a HUD microscope was performed in 47 consecutive procedures and compared with a control group who underwent surgery with classic MTS. In the surgeons’ experience, enhanced AR visualizations improved their three-dimensional perception compared to the standard display, particularly in a case of recurrent Cushing’s disease, where intuitive AR-assisted surgical orientation has simplified the removal of scar tissue and allowed for an easier localization of the recurrent adenoma. Carl et al. demonstrated that the overall clinical accuracy of the AR application depends on the navigation accuracy and on microscopic calibration, indeed the TREs ranged from 0.55 to 4.78 mm (in the fiducial-based registration group) and from 0.21 to 2.07 mm (in the iCT-based registration group). A similar study and results were carried out by Bopp et al. [28], in which 81 patients underwent surgery with classic MTS while 84 patients underwent surgery with HUD-microscope-supported TS. The overall clinical accuracy of the AR application, even in this case, significantly increased in the iCT-based registration group (TRE, 0.76 ± 0.33 mm) compared to the fiducial-based registration group (TRE 1.85 ± 1.02 mm). The application of AR clearly enhanced the intraoperative orientation in the aforementioned cases, especially when intraoperative landmarks were missing due to previous surgery or to particular anatomical variants; it contributed to patient safety and also increased the surgeon’s comfort [28]. Moreover, Cabrilo et al. have attempted to demonstrate the usefulness and ease of application of augmented reality-based neuronavigation through a case example of a recurrent clival chordoma; unlike previous cases, they reported how the use of microscope-based AR overlays 3D images directly onto the microscope’s eyepiece, guiding the surgeon to find a cleavage plane among multilayer fibrosis from previous surgeries [20].

#### 4.2.2. Augmented Reality’s Application in Endoscopic Transsphenoidal Surgery (ETS)

Endoscopic transsphenoidal skull base surgery requires a simultaneous picture of the relationship between the lesion and the surrounding anatomical structures, including cranial nerves and critical vascular structures, from the limited landmarks enclosed in the sphenoidal sinus; therefore, Image-Guided Surgery (IGS) systems are often used to facilitate the real-time anatomical orientation of these structures [42,43]. However, there may be a mismatch between the anatomical topography in the surgical field and the 2D images on the navigation monitor, as described above for conventional navigation systems. In order to deal with this, over the past few decades, AR has also been explored as a tool to improve endoscopic navigation. Indeed, AR can be used to augment the live video stream from the endoscope by superimposing computer-generated image data from pre- or intra-operative radiological examinations, such as MRI or CT scans, onto the real-world view of the endoscope [43]. Most of the studies included in this systematic review investigated the potential role of AR in ETS as superimposing 3D virtual objects on the endoscopic view [1,17,19,21,22,25,26,27].

### 4.3. AR’s Application in ETS (Cadavers or Experimental Models)

Some studies have evaluated the accuracy of the different AR-assisted neuronavigation systems previously on cadaver specimens or experimental models (i.e., phantoms) before testing them on real patients. For instance, to evaluate the accuracy and performance of some AR-assisted navigation systems during ETS, Bong et al. and Lai et al. have used a phantom to simulate the workflow that might occur in a real surgical scenario [22,25]. In the first case, Bong’s group [22] experienced the use of AR-assisted ETS with an optical system for endoscopic calibration and tracking during two sets of experiments, firstly measuring the spatial errors of a virtual object superimposed on the endoscopic view, and secondly performing simulated surgical tasks using a phantom model to compare the performance of the AR navigation system with the conventional endoscopic procedure. Using the AR neuronavigation system, they showed that the mean spatial errors of the AR were ~1 mm and also that the number of error events decreased from 4.86 ± 1.22 to 1.71 ± 1.38 (*p* = 0.0073 < 0.05), and the duration of error events decreased from 2.00 ± 0.75 s to 0.58 ± 0.61 s (*p* = 0.0013 < 0.05), with statistically significant differences. Nevertheless, even if the mean total operation time was slightly longer with the AR-based navigation system (18.26 ± 7.82 s) than with the conventional endoscopic method (13.75 ± 6.30 s), these experiments have shown that AR guarantees a more precise and safer performance. In the second case, an augmented reality surgical navigation system (ARSN) with 3D cone beam computed tomography (CBCT) superimposed on the endoscope’s view was used by Lai et al. [25]. Even in this case, the navigation system included an optical tracking system (OTS) with four video cameras, but these were embedded in the flat detector of the motorized C-arm for image acquisition. The accuracy of the CBCT image’s co-registration was tested using a custom-made grid with embedded 3D spheres and the overall TRE was 0.55 ± 0.24 mm, with a median of 0.51 mm and an interquartile range of 0.39–0.68 mm. In either case, the spatial errors were in within the acceptable range for skull base surgery [39]. Instead, Li et al. [21] conducted experiments both on a skull phantom and a cadaver to determine the display effect and accuracy of their AR-assisted navigation system. Indeed, in the first phase, they used a qualitative polyvinyl chloride (PVC) skull phantom with a top that can be opened and six non-coplanar reference markers (located in the parietal, frontal, temporal and mastoid processes regions bilaterally) to perform a CT scan to obtain imaging data. Then, nine anatomical markers were identified with a calibrated probe and then transferred to the workstation to obtain 3D coordinates. The spatial distance between the two coordinates (the virtual image and the corresponding position) was calculated as the target registration error (TRE), so that the average TRE of the system was 1.19 ± 0.42 mm. Secondly, they performed a cadaver head experiment that demonstrated the effectiveness of the display effect of the AR-assisted navigation system in an anatomical and structural environment. Indeed, a comparison of the performance of the AR-assisted navigation system with the conventional one during the simulated surgeries has shown that the average TRE rates were 1.28 ± 0.45 and 1.32 ± 0.41 mm, respectively, and the average OT was 88.27 ± 20.45 and 104.93 ± 24.61 min, respectively, demonstrating the superiority of the AR-assisted neuronavigation system in terms of timing as well, contrary to what Bong et al. have previously suggested [22]. With regard to cadaver dissection studies, two consecutive studies by Dixon et al. [18,19] using AR-assisted ETS (an ART-IGS system) on cadaver specimens showed that there was a significant decrease in the mental and temporal demand, effort and frustration of surgeons, as measured by the National Aeronautics and Space Administration Task Load Index (NASA-TLX), in the ART-IGS group compared to the control group (*p* < 0.02). In all cases, the TRE was between 1 mm and 1.8 mm. Furthermore, Prisman et al. [17] practiced surgical tasks such as uncinectomy, ethmoidectomy, sphenoidectomy/pituitary resection and clival resection on a cadaveric specimen during an endoscopic surgical approach to the skull base. They found that the preoperative contouring of neurovascular structures, combined with the ability to control the distance of the surgical plane from the tip of the endoscope, was particularly useful, with a mean calculated TRE < 2 mm. Even in these cases, all cadaver dissection studies were well below the currently accepted TRE of 2.0 mm and were also in line with the goal of Citardi et al. [35], who stated that the TRE target for a next-generation surgical navigation platform would be 1.0–1.5 mm, and ideally 0.6–1.0 mm [35,36].

### 4.4. AR’s Application in ETS (Vivo)

Once the accuracy of AR-assisted ETS systems has been assessed in both phantom and cadaveric experimental models, it will be possible to assess its accuracy in vivo. In the studies included in this systematic review, AR-assisted ETS systems were found to be particularly useful in cases where the classic anatomy had been compromised, or even in children, where incomplete pneumatization of the sphenoid sinus, bone thickness and distance between the carotid arteries may affect surgical planning. In such cases, endoscopic-assisted skull base surgery combined with navigation systems is even more important. Indeed, Kawamata et al. [15] used an endoscopic AR-assisted navigation system, in which 3D virtual images of 12 consecutive pituitary tumors and nearby anatomic structures were superimposed on real-time live endoscopic images. In their experience, the colors of the wireframe images of the tumor change according to the distance between the tip of the endoscope and the tumor, giving the surgeon an accurate current orientation and warning of delicate structures. The error of the superimposed wireframe images was always less than 2 mm. In addition, a few studies have investigated and evaluated the use of this type of advanced technology in pediatric case series. First Caversaccio et al. [16] and then Pennacchietti et al. [27] reported their first experience with AR-assisted navigation in endoscopic skull base surgery, even in a pediatric case series. In their experience, simultaneous visualization of the endoscope position on standard axial, sagittal and coronal MRI views, as well as the trajectory-aligned reconstruction of the 3D MRI images on the navigation screen, was able to overcome the morphological anatomical variability in all pediatric cases. Finally, Zeiger et al. described the first clinical series of complex skull base pathologies treated using a novel mixed reality platform (EndoSNAP, Surgical Theatre, Mayfield Village, Ohio). Firstly, 3D digital models of the patient’s anatomy using EndoSNAP were added to Brainlab’s Cranial Navigation software (Brainlab, Munich, Germany), and then fiducials markers were added to the endoscope. A dynamic image of the 3D reconstruction was displayed alongside a corresponding endoscopic camera view, matching the real view with the patient’s anatomy reconstruction. Although the accuracy of the system was not quantified, surgeons found EndoSNAP particularly helpful in cases where atypical pathologies or altered anatomical relationships were found [26].

## 5. Limitations and Future Directions

In the current systematic review, augmented reality-assisted transsphenoidal surgery (TS) has demonstrated significant utility in cadaveric dissection, experimental phantom studies, and in vivo experiments. However, the presence of diverse studies utilizing different augmented reality techniques in both microscopic and endoscopic TS may have impacted our conclusions. Notably, most studies were not in vivo, potentially leading to an exaggerated perception of the technology’s efficiency and effectiveness. Moreover, these studies failed to clearly define their prognosis and outcomes.

The transition from successful controlled experiments to practical clinical applications is challenging due to patient variability and the complexities of real-time surgery. While most studies in this review achieved an acceptable target registration error, a limitation is the scarcity of studies comparing the accuracy of AR-assisted neuronavigation with conventional methods, i.e., lacking a ‘control group’. Consequently, we cannot definitively establish the superiority of AR-assisted neuronavigation over conventional approaches.

Therefore, considering our achievements and the highlighted limitations, further research, especially in vivo studies, is imperative to accurately determine the efficiency of applying this advanced technology in TS surgery, particularly in the context of neuronavigation-based transsphenoidal surgery. Additionally, a comprehensive validation of its safety and efficacy, along with the establishment of a standardized regulatory framework, is essential for its reliable use in future medical settings.

## 6. Conclusions

In order to answer to our initial question, our systematic review provides a comprehensive analysis of the available literature to define the potential role of AR in both endoscopic and microscopic transsphenoidal surgery. Although the reported data were derived from the analysis of heterogeneous studies, including in vivo studies, cadaver dissection studies and also experimental studies, we found that AR navigation and guidance have proven to give added value to standard navigation in transsphenoidal surgery. Indeed, by creating an immersive virtual world, the ‘neuroverse’, in which real-time AR information including 3D reconstructions of the surgical field, anatomical landmarks and tumor boundaries are superimposed on the surgeon’s view, AR-assisted TS, including AR-assisted MTS and, the most widely used, AR-assisted ETS, have been shown to be associated with improved reported outcomes in terms of landmark identification, intra-operative navigation and the surgeon’s perspective, compared to their non-AR counterparts, especially in patients with significant anatomical variations. Finally, AR-assisted neuronavigation has been shown to improve the surgeon’s visualization, spatial orientation and accuracy during neurosurgical procedures and, once perfected and tested in more in vivo studies, may represent a truly revolutionary technique for transsphenoidal surgery.

## Figures and Tables

**Table 1:** Included studies evaluating the use of AR in transsphenoidal surgery.

Authors	Study Design	Nr of Patients and Sex	Age	Test Subjects	Pathology	Landmark Identification	AR Display Device(s)	AR Technique	Accuracy Results	Surgical Time	Reoperation or Anatomical Variants	Outcomes	AR Surgeons’ Perspective
Kawamata et al. 2002 [15]	Technical note	12	N/S	Vivo	Pituitary adenomas (9), craniopharyngioma (1), Rathke’s cleft cyst (1), chordoma (1)	Internal carotid arteries, optic nerves, sphenoid sinuses, sphenopalatine foramen midline, vidian nerve, pituitary gland	Pentium III-based personal computer	Virtual 3D anatomical images overlaid on live endoscopic images	TRE was less than 2 mm	N/S	N/S	N/S	Surgeons can define the exact real-time surgical orientation
Caversaccio et al. 2007 [16]	Retrospective cohort study	313	N/S	Vivo	Recurrent polyposis nasi (Widal), chronic sinusitis (181), biopsy (29), frontal sinus recess revision (29), tumor (22) sphenoid sinus (fungi) (18), mucocele (11), choanal atresia (8), CSF leak (7), recurrent cystic fibrosis (6), embolization (1), crista galli cyst (1)	N/S	Color-coded AR images superimposed onto the endoscopic or microscopic view	Virtual 3D anatomical images overlaid on live endoscopic or microscopic images	1.1–1.8 mm accuracy for position detection	Reduces surgery time by 10–25 min	Recurrent cystic fibrosis (6)	An improvement in the quality of patient outcomes was observed compared to the control group and the literature	N/S
Lapeer et al. 2008 [1]	Original article	N/S	N/S	Experimental model	N/S	N/S	AR images superimposed onto the endoscopic view	Virtual 3D anatomical images overlaid on live endoscopic images	The TRE near ROI was 2.3 ± 0.4 mm	N/S	N/S	N/S	N/S
Prisman et al. 2011 [17]	Original article	3	N/S	Cadaver dissection	N/S	Pituitary gland turbinate, hiatus semilunaris, infundibulum, ethmoid bullae, agger nasi, frontal recess, fovea ethmoidalis, cribriform, lateral papyracea, sphenoethmoid recess, infraorbital nerve, sella	AR images superimposed onto the endoscopic view	Virtual 3D anatomical images overlaid on live endoscopic images	TRE ≤ 2 m	N/S	N/S	N/S	AR was defined as a useful tool for all surgical tasks, especially in the spatial understanding of anatomical structures
Dixon et al. 2012 [18]	Original article	N/S	N/S	Cadaver dissection	N/S	Internal carotid arteries, optic nerves, pituitary gland, pterygopalatine fossa, periorbital fossa	AR images superimposed onto the endoscopic view	Virtual 3D anatomical images overlaid on live endoscopic images	TRE was 1–2mm	The system was considered sufficiently accurate by most surgeons	N/S	N/S	Mental demand, time demand, effort and frustration were significantly reduced
Dixon et al. 2013 [19]	Randomized-controlled trial plus qualitative analysis	14	N/S	Cadaver dissection	N/S	Internal carotid arteries, optic nerves, pituitary gland, orbits	AR images superimposed onto the endoscopic view	Virtual 3D anatomical images overlaid on live endoscopic images	TRE were in line with current clinical practice < 2 mm	N/S	N/S	N/S	Mental demand, time demand, effort and frustration were significantly reduced
Cabrilo et al. 2014 [20]	Technical note	1 M	46 years	Vivo	Recurrent inferior clivus chordoma	Tumor, internal carotid arteries, vertebral arteries	AR images superimposed onto the microscope’s ocular	Virtual 3D anatomical images overlaid on live microscopic images	N/S	N/S	Fibrosis from previous surgery, making it difficult to assess the depth of the tumor	N/S	It reduces the surgeon’s need to mentally match neuronavigation data to the surgical field.
Li et al. 2016 [21]	Research article	N/S	N/S	Cadaver dissection and Experimental model	N/S	Piriform aperture vertex, bilateral supraorbital foramen, turbinate, anterior and posterior clinoid process	AR images superimposed onto the endoscopic view	Virtual 3D anatomical images overlaid on live endoscopic images	TRE was 1.28 ± 0.45 mm	88.27 ± 20.45 for AR-neuronavigation system vs. 104.93 ± 24.61 min for the conventional one	N/S	N/S	Mental and time demand, such as effort and frustration, were significantly reduced
Bong et al. 2018 [22]	Original article	N/S	N/S	Experimental model	N/S	N/S	AR images superimposed onto the endoscopic view	Virtual 3D anatomical images overlaid on live endoscopic images	TRE was <1 mm	Total operation time was lightly longer with the AR system (18.26 ± 7.82 s) than in the control group (13.75 ± 6.30 s).	N/S	N/S	Surgeons performed the task with less mental effort
Barber et al. 2018 [23]	Short scientific communication	1 F	48 years	Vivo	Petrous apex cyst (1)	internal carotid arteries, jugular bulb, facial nerve, petrous apex cyst, cochlea, semicircular canal, sigmoid sinus	AR app built for Android mobiles	3D-printed CT scans were imported onto a the Stealth3D workstation	The range of error between landmarks was 0.7 mm	N/S	N/S	Patient experienced symptomatic relief one year postoperatively	AR support allows surgeons to avoid critical structures
Carl et al. 2019 [24]	Original article	47 (28 M, 19 F)	55.25 (range 16–85 years)	Vivo	Recurrent inactive adenoma (14), recurrent Cushing disease (3), acromegaly, kissing carotid arteries (3), Rathke cyst, incomplete pneumatization (3), recurrent Rathke cyst (3), recurrent acromegaly (2), Inactive macroadenoma (2), recurrent craniopharyngioma (1), cavernous sinus meningioma (1), Cushing disease, incomplete pneumatization(1), recurrent prolactinoma (1), clival ependymoma (1), recurrent pleomorphic sarcoma (1), clival fibrous tumor (1), craniopharyngioma (1), sphenoidal adenoid cystic carcinoma (1), sphenoidal aspergilloma and intracavernous aneurysm (1), fibrous dysplasia (1), CSF fistula after adenoma resection (1), clival lipoma (1), sphenoidal giant inactive adenoma (1), adenoma, Addison (1), sphenoidal giant inactive adenoma (1), sphenoidal prostate carcinoma metastasis (1)	Internal carotid arteries, skull base, optic nerve	Heads-up microscopic display	AR was established using the heads-up displays integrated into operating microscopes	The TRE in patients with fiducial-based registration was 2.33 ± 1.30 mm. The TRE in the iCT-based registration group was 0.83 ± 0.44	N/S	28 reoperations	AR was reported to increase intraoperative orientation markedly	AR support increases surgeon security and awareness
Lai et al. 2020 [25]	Research article	N/S	N/S	Experimental model	N/S	Two inserts simulating the internal carotid arteries, one insert simulating the optic nerve and one insert to simulate the pituitary gland	AR images superimposed onto the endoscopic view	Virtual 3D anatomical images overlaid on live endoscopic images	TRE was 0.55	N/S	N/S	N/S	N/S
Zeiger et al. 2020 [26]	Retrospective cohort study	134 (64 F, 70 M)	56.4 ± 14.6 years	Vivo	Nonsecretory pituitary tumor (53), secretory pituitary tumor (15), meningioma (16), Rathke’s cyst (10), chronic rhinosinusitis (7), chordoma (3), CSF leak (3), epidermoid cyst (3), olfactory neuroblastoma (3), pituitary cyst (2), craniopharyngioma (2), mucocele (2), encephalocele (2), squamous cell carcinoma (2), inverted papilloma (1), juvenile nasopharyngeal angiofibroma (1), acute invasive fungal sinusitis (1), malignant mixed germ cell tumor (1), myxoid chondrosarcoma (1), nasopharyngeal carcinoma (1), plasmacytoma (1), sarcoid (1), pituitary abscess (1), ectopic pituitary tumor (1), sinonasal sarcoma (1)	Internal carotid arteries, vomer, sphenoid, optico-carotid recess	AR images superimposed onto the endoscopic view using EndoSNAP	Virtual 3D anatomical images overlaid on live endoscopic images using EndoSNAP	Surgeons reported that the system was accurate in almost all cases.	Mean operative time was 3 h and 48 min ± 2 h and 3 min	N/S	Hospital re-admission (6.7%), hyponatraemia (6.5%), DI (6.5%)	AR support allows surgeons to identify the delicate structures in order to properly preserve them
Pennacchietti et al. 2021 [27]	Case series	11 (6 M, 5 F)	14.5 ± 2.4 years	Vivo	Craniopharyngio (1), Rathke’s cysts (3), GH-secreting macroadenoma (1), myxoma (1), germinoma (1), aneurysmal bone cyst (1), CSF leak (1), benign fibrous lesion (1), osteochondromyxoma (1)	Internal carotid arteries, optic nerves, tuberculum sellae, optico-carotid recesses, dorsum sella	AR images superimposed onto the endoscopic view.	Virtual 3D anatomical images overlaid on live endoscopic images	N/S	Mean duration of surgery was 146.7 ± 52.6 min (range: 27–258 min)	Craniopharyngioma (4 surgeries), Myxoma (3 surgeries) and 1 recurrent Rathke’s cleft cyst	Complete remissions (8), progressive craniopharyngioma (1), stable diseases (4), panhypopituitarism (3)	AR-assisted neuronavigation was found to be extremely helpful with its approach guidance
Bopp et al. 2022 [28]	Prospective cohort study	84 (41 M, 43 F)	55.95 ± 17.65 years	Vivo	PitNET (2), not specified (82)	Tumor, internal carotid arteries, chiasm, optic nerves	Heads-up microscopic display	Virtual 3D anatomical images overlaid on live microscopic images	The TRE was 0.76 ± 0.33 mm	The mean surgery time was 69.87 ± 24.71 min. The patient preparation time was 44.13 ± 13.67	N/S	Intraoperative CSF leakage (42.86%), major complications (0%), postoperative CSF fistula (3.57%)	AR significantly and reliably facilitated surgical orientation, increasing surgeons’ comfort and patient safety, especially for anatomical variants
Goto et al. 2023 [29]	Original article	15 (8 M, 7 F)	55.88 (range 16–82 years)	Vivo	Petroclival meningioma (2), NF-PitNET (5), PitNET (1), skull base chondrosarcoma (2), intraorbital cavernous malformation (1), craniopharyngioma (1), chordoma of the craniovertebral junction (1), meningioma of the tubercle sellae (1), somatotroph-PitNET (1)	Tumor, internal carotid arteries, sphenoid sinus, clival recess, optic canals and carotid prominence, and the saddle floor	3D images were superimposed onto the second monitor in preparation for the topographic adjustment.	Virtual 3D anatomical images overlaid on live endoscopic images using EndoSNAP	N/S.	N/S	N/S	N/S	AR support was superior to conventional systems in providing a direct 3D view of the tumor and surrounding structures

AR = Augmented Reality; N/S = Not Specified; CSF = cerebrospinal fluid; PitNET = Pituitary Neuro Endocrine Tumors; NF-PitNET = Non-Functioning Pituitary Neuro Endocrine Tumors; TRE = target registration error; FRE = Fiducial Registration Error; ROI = Region Of Interest; iCT = intraoperative Computed Tomography.

## Data Availability

Not applicable.
